# NDRG1 inhibition sensitizes osteosarcoma cells to combretastatin A-4 through targeting autophagy

**DOI:** 10.1038/cddis.2017.438

**Published:** 2017-09-14

**Authors:** Hongsheng Wang, Wen Li, Jing Xu, Tao Zhang, Dongqing Zuo, Zifei Zhou, Binhui Lin, Gangyang Wang, Zhuoying Wang, Wei Sun, Mengxiong Sun, Shimin Chang, Zhengdong Cai, Yingqi Hua

**Affiliations:** 1 Department of Orthopaedics, Yangpu Hospital, Tongji University, Shanghai, China; 2 Department of Orthopaedics, Shanghai General Hospital, School of Medicine Shanghai Jiao Tong University, Shanghai, China; 3 Department of Oncology, Municipal Hospital of Traditional Chinese Medicine, Shanghai University of Traditional Chinese Medicine, Shanghai, China; 4 Shanghai Bone Tumor Institution, Shanghai, China

## Abstract

Combretastatin A-4 (CA-4), a tubulin-depolymerizing agent, shows promising antitumor efficacy and has been under several clinical trials in solid tumors for 10 years. Autophagy has an important pro-survival role in cancer therapy, thus targeting autophagy may improve the efficacy of antitumor agents. N-myc downstream-regulated gene 1 (NDRG1) is a significant stress regulatory gene, which mediates cell survival and chemoresistance. Here we reported that CA-4 could induce cell-protective autophagy, and combination treatment of CA-4 and autophagy inhibitor chloroquine (CQ) exerted synergistic cytotoxic effect on human osteosarcoma (OS) cells. Meanwhile, CA-4 or CQ could increase the expression of NDRG1 independently. We further performed mechanistic study to explore how CA-4 and CQ regulate the expression of NDRG1. Using luciferase reporter assay, we found that CA-4 transcriptionally upregulated NDRG1 expression, whereas CQ triggered colocalization of NDRG1 and lysosome, which subsequently prevented lysosome-dependent degradation of NDRG1. Further, we showed that knockdown of NDRG1 caused the defect of lysosomal function, which accumulated LC3-positive autophagosomes by decreasing their fusion with lysosomes. Moreover, NDRG1 inhibition increased apoptosis in response to combination treatment with CA-4 and CQ. Taken together, our study revealed abrogation of NDRG1 expression sensitizes OS cells to CA-4 by suppression of autophagosome–lysosome fusion. These results provide clues for developing more effective cancer therapeutic strategies by the concomitant treatment with CA-4 and clinical available autophagy inhibitors.

Autophagy is an evolutionarily conserved, homeostatic process that components of the cell are degraded to maintain essential activity and viability as a response to numerous stimuli.^1^ Autophagy begins with the formation of double-membrane autophagic vesicles (AVs), known as autophagosomes, which engulf damaged or superfluous proteins and organelles. The autophagosomes subsequently fuses with lysosomes form the autolysosomes (signal-membrane AVs), where the components inside are degraded and recycle. Because of autophagy major role in cell survival during unfavorable conditions, targeting autophagy may be a reasonable anticancer strategy that improves the efficacy of many standard of care agents. Consistent with this viewpoint, growing evidence shows that autophagy inhibitors like chloroquine (CQ) or 3-methyladenine (3-MA) sensitize cancer cells to chemotherapy treatments like DNA-damage agent doxorubicin,^2^ DNA alkylating agent cisplatin,^3^ microtubule-targeting agent vincristine,^4^ anti-angiogenic agent bevacizumab^5^ and tyrosine kinase receptor inhibitor imatinib.^6^ Hence, understanding how autophagic machinery regulates chemotherapy sensitivity is crucial for cancer therapy.

Combretastatin A-4 (CA-4), a tubulin-depolymerizing agent, shows a great effect in antitumor therapy and has entered clinical trials of solid tumors over 10 years. CA-4 phosphate (CA-4P) is a water-soluble CA-4 prodrug. CA-4 has a high affinity for tubulin, and destabilizes the tubulin polymers of the cytoskeleton, resulting in morphological changes. These changes increase vascular permeability and disrupt tumor blood flow.^7, 8^ Anti-vascular effects *in vivo* are seen within minutes of drug administration and rapidly lead to extensive ischemic necrosis in areas that are often resistant to conventional anticancer treatments.^9, 10^ Recently, increasing evidence has implicated that suppression of autophagy has been suggested to potentially enhance the therapeutic efficacy of CA-4.^11, 12^ Nevertheless, whether disrupting autophagy would augment the anticancer activity of CA-4 in osteosarcoma (OS) cells is still unknown and needs further clarification.

The N-*myc* downregulated gene 1 (NDRG1) is a member of the NDRG family, which belongs to the *α*/*β* hydrolase superfamily, and overexpressed in several types of human carcinomas.^13^ Most intensive studies indicated that the function of NDRG1 is associated with inhibiting cancer metastasis and progression in cancer of brain, breast, colon, rectum, esophagus, pancreas and prostate.^14, 15, 16^ Paradoxically, it has been suggested to promote vascular invasion, metastasis and poor prognosis in cancers of the kidney, liver, mouth, skin and uterine cervix.^17, 18^ Collectively, NDRG1 has an important role of promoting or inhibiting in cancer patients depending upon the tumor species, histological type and differentiation status of human malignancies.^19^ NDRG1 is also recognized as a significant stress response gene and is regulated by a wide range of stress stimuli, such as hypoxia, homocysteine, nickel, androgens, calcium and iron depletion, and chemotherapy.^20^ Recently, studies have been suggested that NDRG1 is involved in modulating sensitivity and resistance of cancer cells to chemotherapeutic agents.^21, 22^ Weiler *et al.*^23^ identified that the mTOR targets NDRG1 as a key determinant of resistance toward alkylating chemotherapy, driven by hypoxia, irradiation, corticosteroids and chronic alkylating agents. Jung *et al.*^24^ also found that hypoxia- and RA-inducible NDRG1 expression is the response for doxorubicin and RA resistance, and the selective interruption of NDRG1 signaling may prove therapeutically useful in hepatocellular carcinoma cells. Hence, these findings indicated that NDRG1 may be developed as an attractive candidate for targeted therapy.

In this study, we investigate the functional and therapeutic relevance of NDRG1 in the combination treatment of CA-4 and autophagy inhibitor CQ against human OS cells. We demonstrated that CA-4 induced cell-protective autophagy, and the combination of CA-4 and CQ exerts synergistic antitumor effects in human OS cells. Meanwhile, both of CA-4 and CQ upregulated the expression of NDRG1 independently. Furthermore, NDRG1 knockdown sensitized human OS cells to CA-4 and CQ combination treatment. Our findings provide clues for developing more effective cancer therapeutic strategies by the concomitant treatment with CA-4 and clinical available autophagy inhibitors.

## Results

### CA-4 induces autophagy in human OS cells

**Figure 1:**
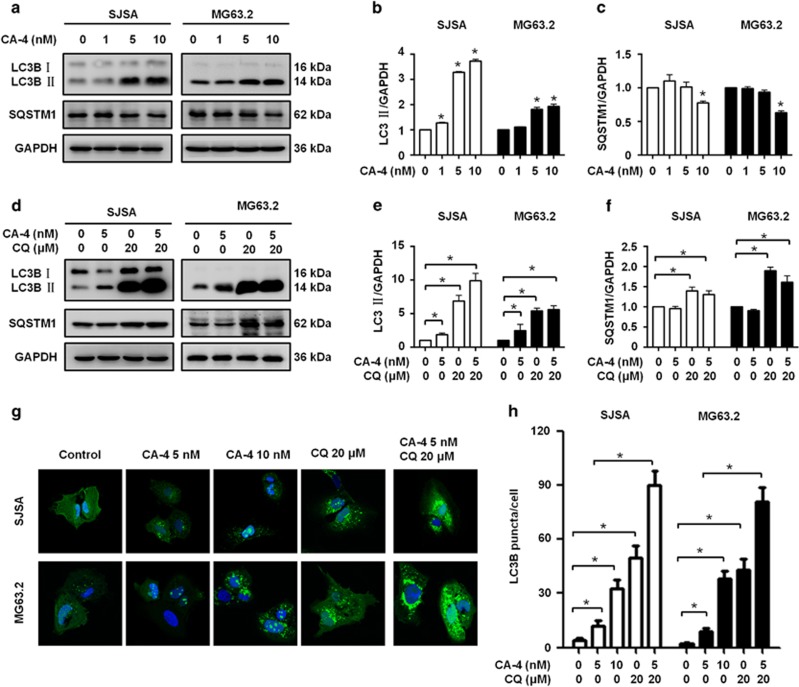
CA-4 induces autophagy in OS cells. (**a**) SJSA and MG63.2 cells were treated with various concentrations of CA-4, and the whole-cell lysates were subjected to immunoblotting of LC3B-II, SQSTM1 and GAPDH. (**b** and **c**) The protein bands in **a** were quantified, and the LC3-II/GAPDH and SQSTM1/GAPDH ratios were calculated and displayed. (**d**) SJSA and MG63.2 cells were treated with CA-4 in the presence or absence of CQ, and the whole-cell lysates were subjected to immunoblotting of LC3B-II, SQSTM1 and GAPDH. (**e** and **f**) The protein bands in **d** were quantified, and the LC3-II/GAPDH and SQSTM1/GAPDH ratios were calculated. (**g**) SJSA and MG63.2 cells expressing GFP-LC3 were treated with control, CA-4 and CQ, and the GFP-LC3 puncta were observed under confocal microscopy. (**h**) Quantification of the number of GFP-LC3 puncta per cell in (**g**). The data were presented as mean±S.D (*P<0.05, *n*=3)

To investigate the effect of CA-4 on autophagy and the role of autophagy in determining the sensitivity of OS cells to this agent, we first examined the activity of autophagy in OS cells treated with CA-4. As shown in Figures 1a and b, OS cells treatment with various concentrations of CA-4 caused a dose-dependent activation of autophagy, as evidenced by a significant increase in the conversion of LC3 from LC3B-I to LC3-II. On autophagic induction, LC3B-I is modified and converted to the phagophore-associated LC3-II through conjugating with the lipid phosphatidylethanolamine. LC3-II levels, thus, serve as an index for the number of autophagosomes.^25^ Similar results were observed in other OS cells lines (Supplementary Figure S1A). LC3-II levels were further elevated in the presence of CQ, a lysosome inhibitor that blocks the fusion of autophagosomes and lysosomes and LC3-II degradation, indicating an increase of autophagic flux in CA-4 treated OS cells (Figures 1d and e). To further evaluate autophagy induction by CA-4 treatment, we established stable cell lines expressing GFP-tagged LC3, representing the formation of autophagosomes (Figure 1g). After quantitation analysis of the number of GFP-LC3 puncta, we observed an accumulation of autophgosomes visualized by a sixfold in CA-4 treatment transfected cells (Figure 1h). In addition, we also examined the SQSTM1 protein level, a selective autophagy receptor protein that interacts with ubiquitinated proteins and LC3-II. The level of SQSTM1 slightly reduced following CA-4 incubation in OS cells (Figures 1a, c, d and f). These findings suggest that CA-4 induces autophagy in OS cells.

### CA-4 and CQ combination treatment has a synergistic effect in OS cells

**Figure 2:**
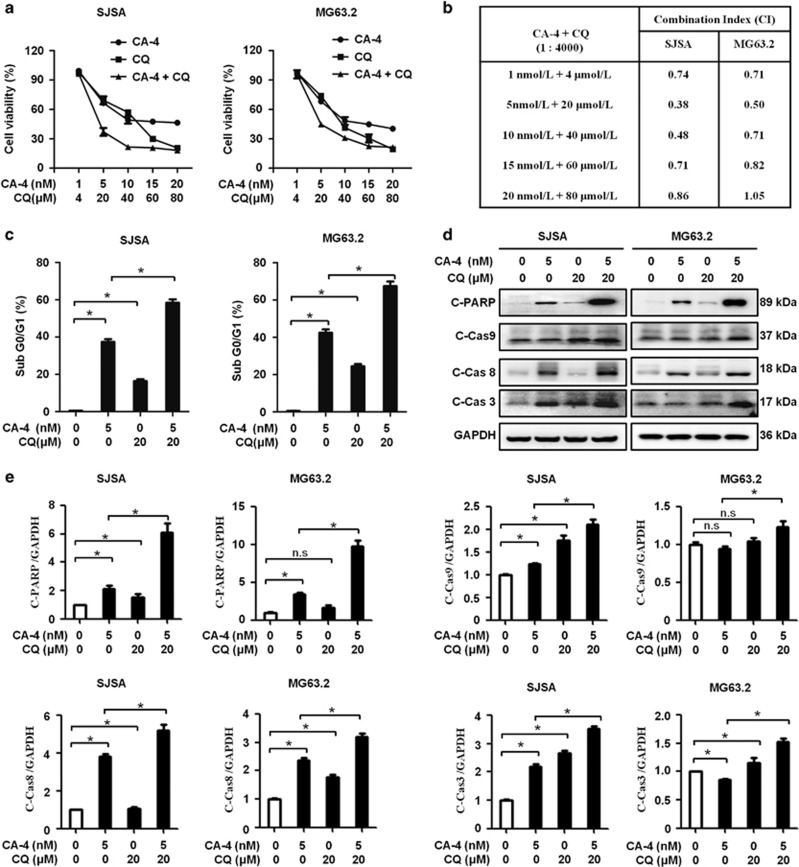
Combination treatment of CA-4 and CQ enhances antitumor activity in OS cells. (**a**) SJSA and MG63.2 cells treated with CA-4 alone, CQ alone, or CA-4/CQ in combination. Cell viability was determined by cell viability assay. (**b**) CA-4/CQ combination induced significantly synergistic effect in SJSA and MG63.2 cells by using CalcuSyn software (Cambridge, UK). (**c**) SJSA and MG63.2 were co-treated with CA-4 and CQ, and apoptosis was indicated as sub-G1 population detected by flow cytometry. Bar graphs showed the percentages of sub-G1. (**d**) Cells were treated as in **c**, and the whole-cell lysates were subjected to immunoblotting of cleaved PARP, cleaved caspases and GAPDH. (**e**) The protein bands in (**d**) were quantified and normalized according GAPDH. The data were presented as mean±S.D (*P<0.05, *n*=3)

To explore whether CA-4-stimulated autophagy is pro-survival or pro-death in OS cells, we used the autophagy inhibitor CQ in combination with this agent. First, we observed a concentration- and time-dependent decrease in cell viability as measured by the CCK8 assay after incubation with a range of CA-4 or CQ at 24, 48 and 72 h (Supplementary Figures S2A and B). We tested the IC_50_ of CA-4 in SJSA and MG63.2 cells at 48 h, and the value was 7.41 and 8.93 nM. The IC_50_ of CQ was 44.26 and 46.96 *μ*M in SJSA and MG63.2 cells at 48 h, respectively. We next examined cell viability following combination treatment with various concentrations of CA-4 and CQ for the cells. Compared with CA-4 or CQ alone, this combined treatment markedly suppressed cell viability (Figure 2a). The combination index (CI) is widely used as an indicator of drug interactions, and quantitative definition of additive effect (CI=1), synergism (CI<1) and antagonism (CI>1). As shown in Figure 2b, the calculated results identified that the combination treatments were synergistic at different combination concentrations especially at lower concentrations. Further experiments with OS cell lines were performed with CA-4 5 nM and/or CQ 20 *μ*M, as these concentrations had the lowest CI reflecting a clear synergistic antitumor activity at 48 h. CA-4 and CQ combination treatment induced a higher percentage of apoptosis compared with each single agent as analysis by FACS after PI staining (Figure 2c). Immunoblotting assay indicated that this combination therapy activated the apoptotic-related proteins PARP and caspases in OS cells (Figures 2d and e). Similar results were obtained in more OS cell lines including CCHO, 143B, K7 and Dunn cells (Supplementary Figure S2C). These results indicated that CQ synergistically enhances the effect of CA-4 on inhibiting cell proliferation and inducing cell apoptosis.

### CA-4 and CQ upregulates NDRG1 expression by different mechanisms, respectively

**Figure 3:**
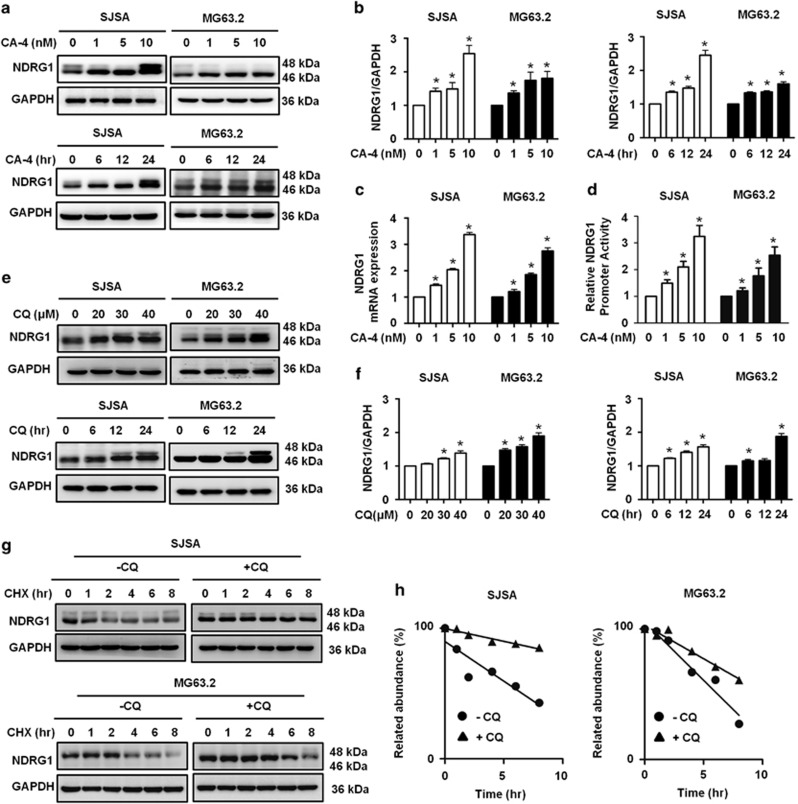
CA-4 and CQ upregulate NDRG1 expression respectively in OS cells. (**a**) SJSA and MG63.2 cells were treated with CA-4, and the whole-cell lysates were subjected to immunoblotting of NDRG1 and GAPDH. (**b**) The NDRG1 double bands in (**a**) were quantified and normalized according GAPDH. (**c**) SJSA and MG63.2 cells were treated with CA-4, and the total mRNA was extracted. Real-time PCR was performed to determine changes in *NDRG1* mRNA. *GAPDH* was used as a loading control. (**d**) The *NDRG1* promoter-driven luciferase reporter was transfected into MG63.2 cells. The results are presented as *NDRG1* promoter activity relative to control (relative *NDRG1* promoter activity). (**e**) SJSA and MG63.2 cells were treated with CQ, and the whole-cell lysates were subjected to immunoblotting of NDRG1 and GAPDH. (**f**) The NDRG1 double bands in (**e**) were quantified and normalized according GAPDH. (**g**) Control and CQ-treated OS cells were exposed to 50 *μ*M CHX, a protein synthesis inhibitor. Cells were harvested at the indicated times (0–8 h) after treatment and analyzed by immunoblotting for NDRG1 and GAPDH. (**h**) NDRG1 levels in (**c**) were quantified and normalized to GAPDH levels, and half-life of NDRG1 was determined by regression analysis. The data were presented as mean±S.D (*P<0.05, *n*=3)

NDRG1 has been recently recognized as a significant stress regulatory gene, which is upregulated by a wide range of stress stimuli.^13, 26^ Nevertheless, it is unknown whether NDRG1 is mediated by CA-4 and CQ in OS cells. First, we examined the effects of CA-4 on expression of in OS cell lines, and results showed CA-4 treatment significantly increased NDRG1 protein levels (Figures 3a and b), and the NDRG1 quantitation included two bands (Figure 3b). We further performed RT-PCR to measure the mRNA level of NDRG1 and found CA-4 treatment induced a significant increase of NDRG1 mRNA level in OS cells (Figure 3c). These results suggested that the regulatory effect of CA-4 on NDRG1 expression may occur at the transcription level. As expected, the full length of the NDRG1 promoter was used to show that CA-4 could concentration-dependently induce luciferase activity (Figure 3d). CQ could disrupt lysosomal structure and function, leading to the inhibition of protein degradation.^27, 28, 29^ This led us to investigate CQ effects on the degradation of NDRG1. We pretreated OS cells with different concentrations of CQ, and results showed that level of NDRG1 protein was markedly increased in a dose-dependent manner by immunoblotting assay (Figures 3e and f), and the NDRG1 quantitation included two bands (Figure 3f). However, pretreatment with CQ did not change NDRG1 mRNA level by RT-RCR assays in OS cells (Supplementary Figure S3A), indicating that CQ-mediated NDRG1 upregulation did not occur at the transcript level, probably because of inhibition of its degradation. To this end, the stability of NDRG1 protein was examined following treatment of cells with protein synthesis inhibitor cycloheximide (CHX). Cells were treated with CHX in the presence or absence of CQ and results showed that the decrease NDRG1 observed after CHX treatment was partially recovered upon co-treatment with CHX and CQ (Figures 3g and h). To further confirm these findings, we identified NDRG1 whose distinct accumulation patterns trapped in lysosomes in the CQ-treated cells (Figure 5a). These results suggested that a lysosome-dependent activity of CQ prevents degradation of NDRG1 protein.

### NDRG1 knockdown causes the accumulation of autophagosomes

**Figure 4:**
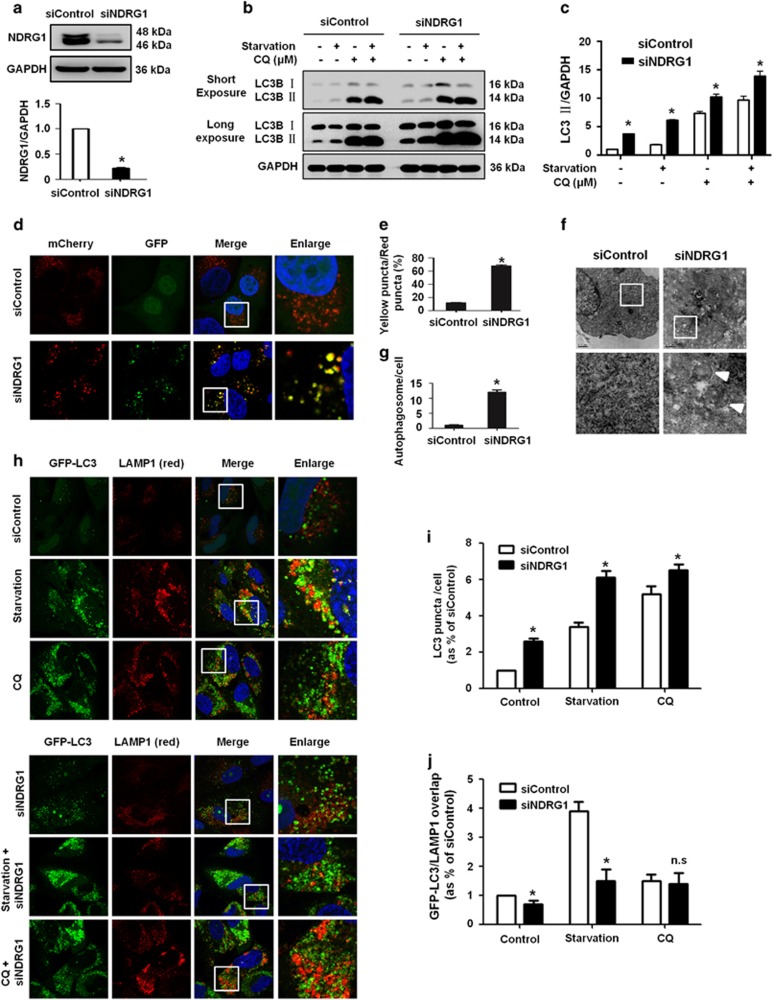
NDRG1 knockdown inhibits autophagosome-lysosome fusion in OS cells. (**a**) MG63.2 cells were transfected with control nontarget or NDRG1 siRNA, and the whole-cell lysates were subjected to immunoblotting. The lower panel showed quantitation of NDRG1 double bands. (**b**) MG63.2 cells transfected with control nontarget or NDRG1 siRNA either cultured under free-serum starved or CQ treatment, and the whole-cell lysates were subjected to immunoblotting of LC3B-II and GAPDH. (**c**) The LC3-II bands in (**b**) were quantified and normalized according GAPDH. (**d** and **e**) MG63.2 cells stably expressing the mCherry-GFP-LC3 reporter were transfected with control nontarget or NDRG1 siRNA. The colocalizations of mCherry and GFP puncta were examined by the confocal microscopy. (**f**) and (**g**) NDRG1 knockdown induced the accumulation of AVs as shown in the electron micrographs. The arrow indicates AVs. (**h**) Control nontarget and NDRG1 siRNA treated MG63.2 cells stably expressing GFP-LC3, and stained with antibodies against LAMP1 for confocal microscopy. (**i**) Quantification of average GFP-LC3 puncta per cell. (**j**) Quantification of GFP-LC3/LAMP1 colocalization co-efficiency. The data were presented as mean±S.D (*P<0.05, *n*=3)

We explored whether there was an association between the function of NDRG1 and autophagy in OS cells. First, we used siRNA to knockdown NDRG1 expression, and overall knockdown efficiency of 80% was confirmed by quantification (Figure 4a). NDRG1 knockdown increased the number of LC3B-II puncta (Figure 4h and Supplementary Figure S4A), and the levels of LC3B-II in OS cells (Figures 4b and c). However, compared with NDRG1 siRNA or CQ treatment alone, combined treatment with NDRG1 siRNA and CQ resulted a slight increase in levels of LC3B- II, suggesting that the basal autophagic flux was increased in NDRG1 silencing (Figures 4b and c). These results were consistent with the previous study, in which NDRG1 was involved in the regulation of stress-induced, pro-survival autophagic pathway.^30^ Furthermore, we examined whether NDRG1 functioned in the late stage of autophagy, tandem mCherry-GFP-LC3 fluorescence assay was used. In this assay, autophagosomes appear as yellow (mCherry and GFP) puncta, whereas autolysosomes appear as red (mCherry) puncta. NDRG1-silencing cells exhibited an accumulation of yellow autophagosomes under basal conditions (Figure 4d). Quantification analysis revealed a significant increase in overlap of the mCherry/GFP fluorescence signal in NDRG1 knockdown cells compared with mock-transfected cells (Figure 4e), indicating that NDRG1 silencing causes impaired fusion between autophagosomes and lysosomes. Transmission electron microscopy was utilized to visualize the ultrastructure of autophagy organelles in OS cells. Large numbers of autophagosomes were observed in NDRG1 knockdown cells maintained in normal culture conditions (Figures 4f and g). Consistently, fluorescent GFP-LC3 puncta were easily observed in NDRG1 knockdown cells (Figures 4h and j), and did not colocalize with lysosome-associated membrane protein 1 (LAMP1) (Figures 4d and k). Collectively, these data suggested that NDRG1 silencing blocks the fusion between autophagosomes and lysosomes, thereby leading to accumulation of LC3B-II-positive autophagosomes.

### NDRG1 knockdown leads to defects in lysosomal activity

**Figure 5:**
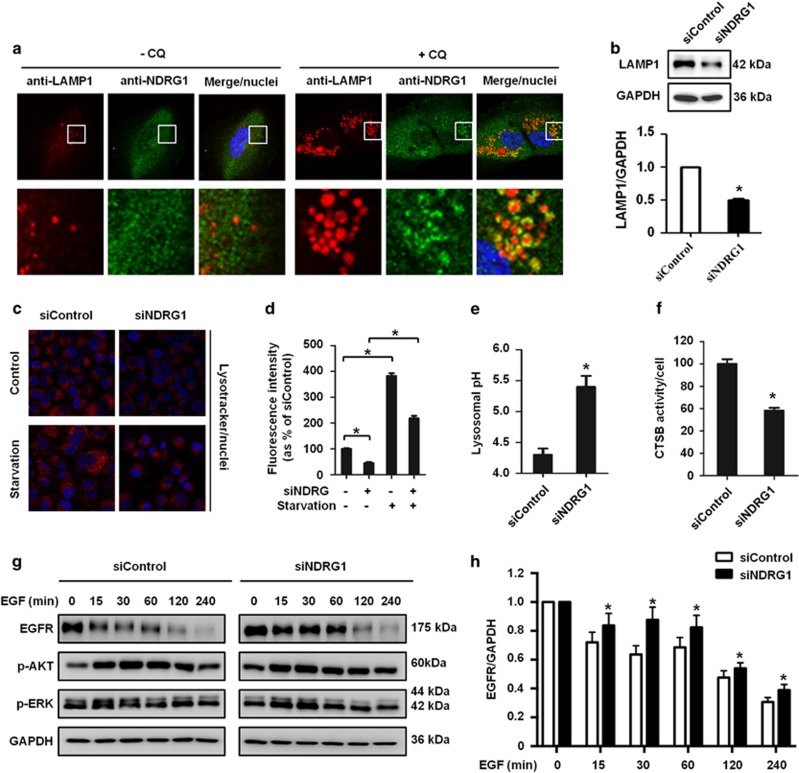
NDRG1 knockdown impairs lysosomal function in OS cells. (**a**) MG63.2 cells were stained with NDRG1 and LAMP1 antibodies in the presence or absence of CQ. The colocalization of NDRG1 and LAMP1 was examined by the confocal microscopy, scale bars: 10 *μ*m. (**b**) Immunoblotting analysis of LAMP1 in MG63.2 cell with NDRG1 knockdown. (**c** and **d**) NDRG1 knockdown interferes with the acidification of lysosomes. MG63.2 cells transfected with control nontarget or NDRG1 siRNA were exposed to LysoTracker for confocal microscopy. (**e**) Lysosomal pH values were measured using a quantitative ratiometric LysoSensor Yellow/Blue DND-160. Scale bars: 500 nm. (**f**) Enzymatic activity of CTSB was measured in MG63.2 cells transfected with control nontarget or NDRG1 siRNA using fluorogenic kits. (**g**) MG63.2 cells transfected with control or NDRG1 siRNA were treated with 50 ng/ml EGF, and EGFR, p-AKT and p-ERK were determined at each point by immunoblotting. (**h**) The EGFR bands in **g** were quantified and normalized according GAPDH. The data were presented as mean±S.D (*P<0.05, *n*=3)

Our data demonstrated that NDRG1 depletion blocks autophagic flux and promotes accumulation of autophagosomes, suggesting that NDRG1 knockdown may impair lysosomal degradation at a point downstream of fusion. We examined the subcellular localization of NDRG1 and LAMP1, a lysosome marker, and results showed the colocalization between NDRG1 and LAMP1 following treatment by CQ (Figure 5a). Furthermore, we investigated the effects of NDRG1 silencing on LAMP1 by immunoblotting, and the results found level of LAMP1 was reduced in NDRG1 knockdown cells (Figure 5b). Based on these findings, we speculated that NDRG1 may be associated with lysosomes. First, we measured the lysosomal acidification, which is required for the maturation and activation of most lysosomal enzymes. OS cells were stained with a specific lysosome dye LysoTracker Red, which accumulates in acidic cellular organelles, and the cells were treated with starvation conditions. We found, compared with nontarget siRNA control cells, the lysosomal fluorescence intensity was lower in NDRG1 knockdown cells (Figures 5c and d,Supplementary Figures S5A and B). We further quantified lysosomal pH using a LysoSensor Yellow/Blue dye, and found that lysosomal pH in NDRG1 knockdown cells was increased from pH 4.3 in control cells to 5.4 (Figure 5e). These results suggested that NDRG1 silencing suppresses the lysosomal acidification. Second, we performed functional assays of lysosomal activity of OS cells. Fluorogenic substrate assay was used to measure the enzymatic activity of CTSB, a member of the cysteine cathepsin family, and results showed that CTSB activity was reduced in NDRG1 knockdown cells (Figure 5f). Finally, we utilized epidermal growth factor receptor (EGFR) as endogenous substrate to examine whether NDRG1 silencing affects the general endosomal–lysosomal clearance. EGFR undergoes endocytosis and degrades in lysosomes. In this assay, OS cells transfected with NDRG1 siRNA were treated with EGF. EGFR downregulation was observed after 15- min treatment with EGF, but NDRG1 inhibition attenuated EGFR clearance. However, there was no obvious change in p-AKT and p-ERK between siControl and siNDRG1 cells (Figures 5g and h). Taken together, these data demonstrated that absence of NDRG1 causes defect in lysosomal activity.

### NDRG1 knockdown sensitizes OS cells to combination treatment CA-4 and CQ

**Figure 6:**
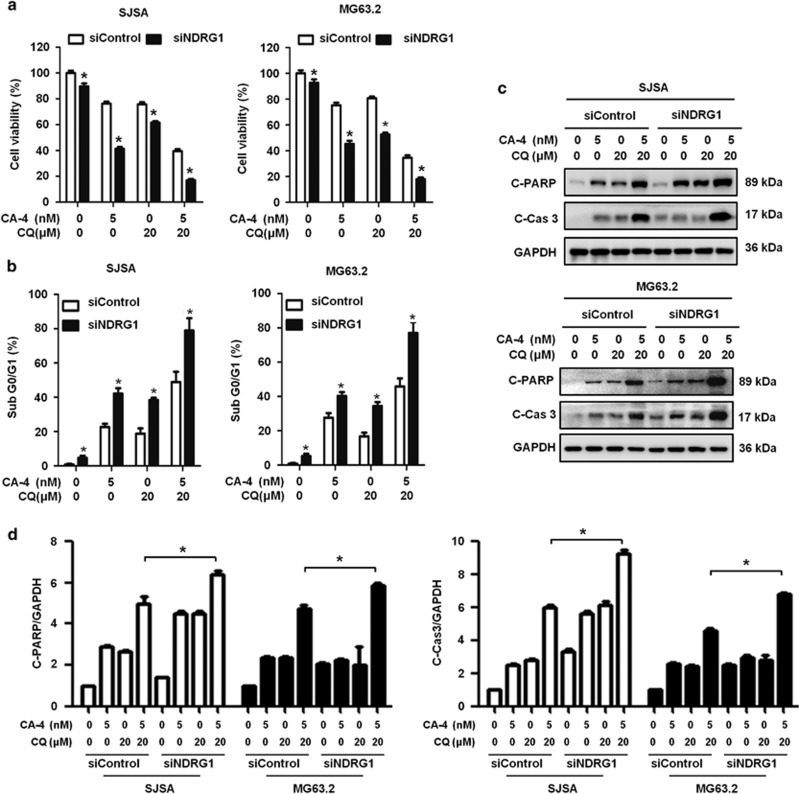
NDRG1 knockdown sensitizes CA-4 and CQ combination treatment-induced apoptosis in OS cells. (**a**) Cells transfected with control nontarget or NDRG1 siRNA treated with CA-4 alone, CQ alone or CA-4/CQ in combination. Cell viability was determined by cell viability assay. (**b**) Apoptosis was indicated as sub-G1 population detected by flow cytometry. (**c**) Cells were harvested, and then the whole-cell lysates were subjected to immunoblotting of cleaved PARP, cleaved caspase 3 and GAPDH. (**d**) The cleaved PARP and cleaved caspase 3 bands in **c** were quantified and normalized according GAPDH. The data were presented as mean±S.D (*P<0.05, *n*=3)

We examined whether the marked increases in NDRG1 expression by the CA-4/CQ regimen had a critical role in the sensitivity of OS cells to apoptosis induced by this combination. Silencing of NDRG1 expression by specific siRNA significantly enhanced the cytotoxicity of the combination of CA-4 and CQ, as compared with those transfected with a non-targeted control siRNA (Figure 6a). We further demonstrated that NDRG1 knockdown significantly increased the CA-4 and CQ-induced apoptosis, as indicated by increases in sub-G0/G1 (Figure 6b), and the amounts of cleaved PARP and cleaved caspase 3 (Figures 6c and d). Suppression of autophagy and activation of apoptosis by inhibiting NDRG1 in the CA-4 and CQ combination treatment cells suggested a regulatory role for NDRG1 in the cross-talk between autophagy and apoptosis. Importantly, these findings established that suppression of NDRG1 increases sensitivity to CA-4 and CQ combination therapy in OS cells.

## Discussion

Autophagy inhibition has been shown to enhance chemosensitivity and antitumor efficacy in multiple tumor cell types, and thus the identification and validation of novel strategies to exploit autophagy for therapeutic advantage against cancer are a significant challenge. We hypothesized that inhibition the autophagy pathway may enhance the antitumor activity of CA-4, and the studies demonstrated that autophagy inhibitor CQ synergistically potentiated the pro-apoptotic effects of CA-4. Furthermore, we also found that both of CA-4 and CQ could increase the expression of NDRG1, a stress response gene that mediates cell survival and chemoresistance. CA-4 transcriptionally upregulated NDRG1 expression, whereas CQ triggered colocalization of NDRG1 and lysosome, which subsequently prevented lysosome-dependent degradation of NDRG1. Silencing NDRG1 suppressed the lysosomal acidification, and impaired function of lysosome endocytic clearance, which leaded to accumulation of autophagosomes. Importantly, inhibition of NDRG1 expression increased apoptosis in response to combination treatment with CA-4 and CQ. Our work demonstrated that NDRG1 linked to chemotherapy sensitivity, and our results may have very important clinical implication.

Chemotherapy-induced autophagy is cyto-protective; consequently, inhibition of autophagy is anticipated to sensitize malignancies to therapy. These findings laid the foundation for clinical and experimental applications combining of autophagy inhibitors (such as CQ) with chemotherapy agents.^31, 32, 33^ CQ, which is a classical anti-malarial and anti-inflammatory drug, inhibits lysosomal acidification and therefore prevents autophagy by blocking autophagosomes fusion and degradation. In cancer treatment, CQ and its derivatives are the few inhibitors of autophagy that are available for use in the clinic, multiple ongoing clinical trials used CQ combination with diverse chemotherapeutic drugs for this purpose.^32, 34^ The vascular targeting agent CA-4 is a microtubule-depolymerizing agent. CA-4P is a water-soluble prodrug of CA-4, and CA-4P is undergoing Phase II and III clinical trials to evaluate the safety and efficacy in human cancer patients when used in combination with chemotherapy or radiation therapy.^35, 36, 37^ In this study, we showed that a combination of CQ could potentiate antitumor effects of CA-4. This finding is also consistent with recent studies, in which CA-4-elicited autophagic response had a protective role that impeded the eventual cell death, whereas autophagy inhibition improved chemotherapeutic efficacy of CA-4.^11, 12^ We further demonstrated that combination of CA-4 and CQ in OS cells synergistically activated the caspase cascade-induced apoptosis, as compared with single-agent treatment at the same concentrations. Moreover, the concentration of each drug administration in the combination was almost 50% less than the IC_50_ of each agent, which favors a tolerability of this combination that will be explored in future clinical trials.

NDRG1 is recognized as a significant stress regulatory gene and is induced by a range of stress, including starvation, hypoxia, homocysteine, nickel, androgens, iron depletion and chemotherapy.^30, 38^ As expected, we demonstrated that CA-4 and CQ treatment could increase the expression of NDRG1, respectively, but with different mechanisms. By using RT-PCR and NDRG1 promoter-driven luciferase reporter, we found CA-4 treatment increased NDRG1 expression at the transcriptional level. As to CQ, our results showed that CQ treatment led to increase of NDRG1 expression and specific colocalization of NDRG1 and lysosomes. Further experiments found that NDRG1 treated by CQ are more rapidly turned over than the absence of CQ using CHX chase experiments. Our mechanistic insight into NDRG1 accumulation by CQ treatment focuses on the lysosome degradation pathway. Interestingly, it appears that the higher band is responding to CQ and CA-4 more markedly than the lower band. The producing of NDRG1 double band has remained controversial. Many studies suggested the higher band is due to NDRG1 phosphorylation.^39, 40^ Indeed, NDRG1 undergoes phosphorylation at multiple sites, resulting in a phosphorylated isoform that is of a higher molecular mass.^41^ However, a study using dephosphorylation assay and Phos-tag SDS/PAGE assay demonstrated that the higher band was not due to phosphorylation, but rather proteolytic cleavage.^42^ In addition, one or more of the NDRG1 bands detected by western blot are due to different NDRG1 primary antibodies or nonspecific binding of either primary or secondary antibodies.^43, 44^ From the above, it is very difficult to speculate the reason of for one or more the NDRG1 bands followed by CQ and CA-4 treatment. It needs further clarification.

Next, we broadened our view to examine the effect of NDRG1 on regulation of autophagy. We observed that loss-of-function of NDRG1 causes an increase in the numbers of autophagosomes, suggesting that NDRG1 silencing may lead to inhibition of subsequent autophagosome process. Our results are in agreement with a recent study, which NDRG1 suppressed iron chelator–mediator accumulation of the autophagic marker LC3-II by activation of the PERK/eIF2α pathway.^30^ The reduction of autophagosomes degradation could be caused by either blocking autophagosomes fusion with the lysosomes, or attenuating the enzymatic activity of lysosome hydrolases.^45, 46^ We found the fusion ability of autophagosomes with lysosomes decreased in NDRG1 knockdown cells. As mentioned above, CQ treatment resulted in NDRG1 colocalized with the lysosomes, therefore we examined whether NDRG1 targeted to lysosomes or alters lysosome function. We found that NDRG1 silencing triggered a reduction in proteolytic activity, and an increase in lysosome pH, indicating that loss of NDRG1 in turn reduced the turnover of autophagosomes. It is noteworthy that poor lysosome activity resulting from defects in lysosomal acidification or maturity had an inhibitory impact on endocytic clearance. EGFR is a typical member of the receptor tyrosine kinase family, which after ligand binding-induced activation is endocytosed and delivered to lysosomes for degradation.^46^ As expected, further experiments showed that a marked defect in ligand-induced EGFR degradation was observed in cells that NDRG1 silencing. Receptor ubiquitylation occurs at the cell surface, its major role is to sort internalized receptors to the lumen of the multivesicular body, en route to the lysosome. Following EGF binding, EGFR sorting to lysosomes depends on its kinase domain and its ubiquitination by Cbl proteins, a cytosolic ubiquitin ligase. Ubiquitination of EGFR is key mechanism in EGF-induced receptor lysosomal degradation. It is a possible mechanism for NDRG1 markedly and significantly inhibiting level and membrane localization of EGFR in a recent study.^47^

NDRG1 activation has frequently been found to promote cancer cell chemoresistance. The molecular mechanism(s) for NDRG1 on chemosensitivity of cancer cells remains unknown. The results of this study imply that autophagy mediated by NDRG1 deficiency via lysosome function impairment may contribute to the sensitivity of cancer cells to chemotherapy drugs. We demonstrated that OS cells transfected with NDRG1 siRNA were significantly higher sensitive to the combination of CQ and CA-4 than those transfected with a non-targeted control siRNA. In contrast to our findings, *Sahni et al.*^30^ reported that NDRG1 upregulation treated by iron chelator–mediator increased the induction of apoptosis in pancreatic cancer cells. This discrepancy may be due to the ability of NDRG1 to regulate autophagy after incubation with iron chelators, which would decrease recycling of nutrients to facilitate apoptotic cell death, as well as differences in the cell types, that is, pancreatic cancer cells *versus* OS cells. Notably, our previous results showed that NDRG1 expression was increased in OS and this elevation was correlated with tumor progression and prognosis,^48^ suggesting that NDRG1 could be considered as a promising therapeutic approach in OS. Therefore, it can be inferred that a combination of NDRG1 inhibition with chemotherapy agents will be used as a useful approach in OS treatment.

**Figure 7:**
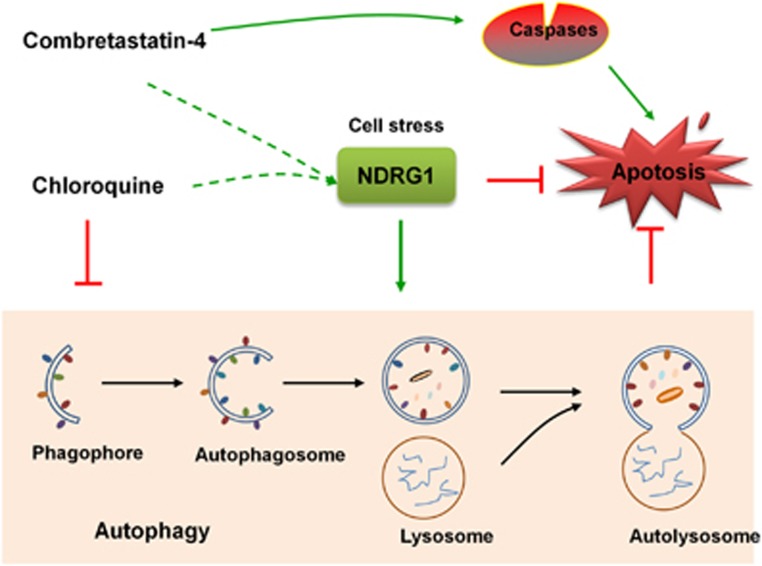
Model of the role of NDRG1 in the CA-4-induced apoptosis and autophagy. Downregulation of NDRG1 expression causes the defect of lysosomal function, subsequently resulting in decreasing the fusion between autophagosomes and lysosomes. Moreover, NDRG1 inhibition increases apoptosis in response to combination treatment with CA-4 and CQ

In summary, our current studies reveal that CA-4 treatment triggers autophagy, and CA-4 and autophagy inhibitor CQ have a synergistic activity against OS cells (Figure 7). In addition, both of CA-4 and CQ upregulated the expression of NDRG1. We also found that the deficiency of autophagy by NDRG1 silencing enhanced the antitumor effect of the combination by impairing lysosome function. Our findings revealed abrogation of NDRG1 function sensitizes OS cells to CA-4 and CQ combination treatment. These results provide clues for developing more effective cancer therapeutic strategies by the concomitant treatment with CA-4 and clinical available autophagy inhibitors.

## Materials and methods

### Reagents and antibodies

The chemicals used in our experiments were: CA-4 (Selleckchem, Houston, TX, USA, S7204), CQ diphosphate (Selleckchem, S4430), and puromycin (Selleckchem, S7417), CHX (Medchem Express, NJ, USA, HY-12320), LysoTracker Red DND-99 (Invitrogen, Carlsbad, CA, USA, L-7258), LysoSensor Yellow/Blue DND-160 (Invitrogen, L-7545). The antibodies used in our experiments were: anti-MAP1LC3B/LC3B (Cell Signaling Technology, Beverly, MA, USA, 2775), anti-SQSTM1 (Cell Signaling Technology, 8025), anti-LAMP1 (Cell Signaling Technology, 9091), anti-NDRG1 (Cell Signaling Technology, 9485), anti-EGFR (Cell Signaling Technology, 4267), anti-p-AKT (Cell Signaling Technology, 13038), anti-p-ERK (Cell Signaling Technology, 9101), anti-cleaved PARP (Cell Signaling Technology, 5625), anti-CDKN1A/p21(Cell Signaling Technology, 2947), anti-CDKN1B/p27 (Cell Signaling Technology, 3686), anti-caspase 3 (Epitomics, Burlingame, CA, USA, 1087-1), anti-caspase 8 (Epitomics, 1007-1), anti-caspase 9 (Epitomics, 3392-1), anti-HIF-1α (Novus, Littleton, CO, USA, NB100-105), anti-CTSB (Proteintech, Rosemont, IL, USA, 12216-1-AP) and anti-CTSD (Proteintech, 21327-1-AP). Alexa Fluor 488- and 568-conjugated secondary antibodies were purchased from Invitrogen (A21429 and A11029).

### Cell lines and culture

Human OS cell lines SJSA, 143B and K7, and mouse OS cell line Dunn were purchased from American Type Culture Collection (ATCC, Manassas, VA, USA). Human OS cell line CCHO was established by Pediatric Research in MD Anderson Cancer Center (Houston, TX, USA). MG63.2 cell was derived from the metastasis of parental MG63, as previously reported.^49, 50^ All cells were finger printed to exclude possible contamination. All cells were cultured in DMEM medium (Thermo, Waltham, MA, USA) supplemented with 10% fetal bovine serum (Thermo), 100 U/ml penicillin and 100 *μ*g/ml streptomycin (Thermo). Cells were maintained at 37 °C in a humidified atmosphere containing 5% CO_2_.

### Plasmid and siRNA transfection

Transfection was achieved using Lipofectamine 2000 Transfection Reagent (Invitrogen, 11668-019) following the manufacturer’s protocol. Cells were transfected with plasmids encoding mCherry-GFP-LC3 (22418) and GFP-LC3 (22405) from Addgene, Cambridge, MA, USA. Human pcDNA3.1(+)-NDRG1 plasmid constructed by Gene-Chem Co. Ltd (Shanghai, China). Human *NDRG1* siRNA, *HIF-1α* siRNA and nontarget siRNA were purchased from Ribobio (Guangzhou, China). Cells were cultured in six-well plates and transfected with plasmids for 24 h. After the designated treatments, live cell images were obtained using a fluorescence microscope (Leica, Wetzlar, Germany). Protein knockdown or overexpression efficiency was assessed by immunoblotting.

### Cell viability assay

The cytotoxic effect of CA-4 on OS cells was determined using the Cell Counting Kit-8 (CCK8) (Dojindo, Kumamoto, Japan). Briefly, cell suspensions (3 × 10^4^/ml) were seeded into 96-well plates overnight and then subjected to different treatments. After 48 h, 10 *μ*l CCK8 solution was added to each well, and the samples were incubated at 37 °C for 2 h before the absorbance was measured at 450 nm wave length.

### Cell apoptosis assay

Apoptosis was evaluated using: (1) flow cytometry via propidum iodide (PI) analysis of sub-G0/G1 DNA content as previous described.^51^ (2) Immunoblotting assay of the cleaved-PAPR and cleaved caspases.

### Immunofluorescence staining and confocal microscopy

Cells were plated on slips, for different experimental conditions, fixed in 4% paraformaldehyde for 15 min at room temperature, and permeablized with 0.1% Triton X-100 (Sigma, St. Louis, MO, USA, 158127). After blocking with 5% BSA (Sangon Biotech Co., Ltd (Shanghai, China), C508113) in PBS for 30 min, cells were incubated with the following primary antibodies: rabbit anti-NDRG1 (1 : 200) and rabbit anti-LAMP1 (1 : 200) in blocking buffer for at 4 °C overnight. The next day, cells were washed and incubated with secondary antibody. Images were obtained using Leica TCS SP5 confocal laser scanning microscope (Leica, Wetzlar, Germany).

### Reverse transcription polymerase chain reaction (RT-PCR)

Total RNA was isolated using Trizol reagent (Invitrogen, 15596018), according to the manufacturer’s instructions. In all, 1 *μ*g RNA was primed with random hexamers and reverse transcribed with PrimerScript Reverse Transcriptase (Takara, Kusatsu, Japan, 6110). The following primers were used: *NDRG1*, forward: 5′-AAGATGGCGGACTGTGGC-3′ reverse: 5′-TCAGGCGGGTCATGCTAG-3′. *GAPDH*, forward: 5′-TGAACGGGAAGCTCA-3′ reverse: 5′-TCCACCACCCTGTTGCTGTA-3′. These primers were synthesized by Sangon Biotech Co., Ltd. All samples were estimated by normalization to *GAPDH* expression levels.

### Immunoblotting

Cells were lysed in RIPA buffer containing protease and a phosphatase inhibitor cocktail from Roche (Indianapolis, IN, USA). A total of 30 *μ*g protein lysates were separated by 6–12% SDS-PAGE and transferred to PVDF membrane from Bio-Rad (Hercules, CA, USA). The membranes were incubated with primary antibody at 4 °C overnight, followed by incubation with peroxidase-conjugated secondary antibody for 1 h at room temperature. The protein signals were detected by ECL from Millipore (Billerica, MA, USA).

### Transmission electron microscopy

Cells were treated as indicated and fixed with 2.5% glutaraldehyde solution (Sigma, St. Louis, MO, USA, G5882) at 4 °C overnight, and post-fixed in 1% buffered osmium tetroxide for 1.5 h at room temperature. The fixed cells were then dehydrated, embedded and stained with uranyl acetate. Representative areas were chosen for ultrathin sectioning and examined by transmission electron microscopy (FEI Tecnai G^2^ 12, Eindhoven, The Netherlands).

### Measurement of lysosome pH

LysoTracker Red DND-99 staining was used to measure the pH of acidic organelles which become more fluorescent in acidic environments, as according to previous protocols.^52^ Briefly, cells from different treatments were incubated with 50 nM of LysoTracker Red DND-99 in the dark for 30 min at 37 °C, and the samples were analyzed by fluorescence microscope or flow cytometry. For lysosome, pH quantification was performed with LysoSensor Yellow/Blue DND-160. Cells were seeded in 96-well plates and labeled with 2 *μ*M LysoSensor for 45 min at 37 °C in regular medium, then washed in PBS. The labeled cells were treated for 10 min with 10 *μ*M monensin (Sigma, M5273) and 10 M nigericin (Sigma, N7143) in 25 mM 2-(*N*-morpholino) ethane sulfonic acid (MES) calibration buffer (5 mM NaCl, 115 mM KCl and 1.2 mM MgSO_4_), which was adjusted with pH from 3.5 to 6.0. The samples were measured with excitation of 335 nm and then the fluorescence emission intensity ratio of 450 nm/520 nm was calculated. The pH value of each sample was determined from the linear standard curve.

### Construction of NDRG1 promoter-driven luciferase reporter

Human NDRG1 promoter was inserted in firefly luciferase reporter vector pGL3-Basic by Longqian Biotech (Shanghai, China). Cells were seeded on 24-well plates, and co-transfected with luciferase reporter vectors and *Renilla* luciferase reporter vector pRL-SV40 (Promega). After 24-h transfection, *firefly* and *Renilla* luciferase activities were consecutively measured, according to the dual-luciferase assay manual (Promega, Madison, WI, USA, E1500). The *firefly* luciferase signal was normalized to the *Renilla* luciferase signal for each individual analysis.

### Statistical analysis

All data were expressed as mean±standard derivation (S.D.) of three independent experiments, as indicated. Statistical significance was assessed by two-sided Student’s *t*-test or ANOVA. Value of *P*<0.05 was considered significant and asterisked without correction for multiple statistical tests.

## Publisher’s Note

Springer Nature remains neutral with regard to jurisdictional claims in published maps and institutional affiliations.
