# Corrigendum: NDRG1 inhibition sensitizes osteosarcoma cells to combretastatin A-4 through targeting autophagy

**DOI:** 10.1038/cddis.2017.558

**Published:** 2017-10-26

**Authors:** Hongsheng Wang, Wen Li, Jing Xu, Tao Zhang, Dongqing Zuo, Zifei Zhou, Binhui Lin, Gangyang Wang, Zhuoying Wang, Wei Sun, Mengxiong Sun, Shimin Chang, Zhengdong Cai, Yingqi Hua

**Correction to:** *Cell Death and Disease* (2017) **8** (9):e3048. doi: 10.1038/cddis.2017.438; published online 14 September 2017

Since the publication of this paper, the authors have noted that there was an error in Figure 1d, in that, the GAPDH for combination treatment with CA-4 and CQ in SJSA cells by mistake duplicated for CQ alone treatment (Figure 3e). This error has now been rectified. The correct figure is shown below.

The authors would like to apologize for any inconvenience this may have caused.

## Figures and Tables

**Figure 1:**
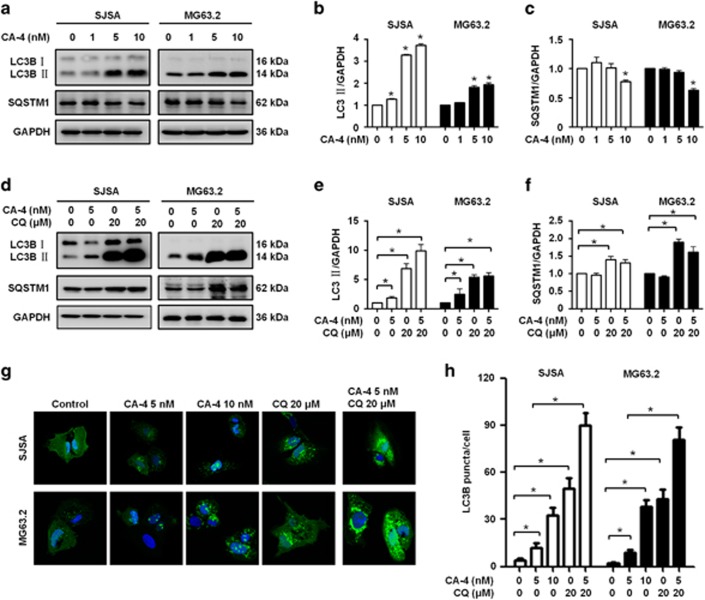
CA-4 induces autophagy in OS cells. (**a**) SJSA and MG63.2 cells were treated with various concentrations of CA-4, and the whole-cell lysates were subjected to immunoblotting of LC3B-II, SQSTM1 and GAPDH. (**b** and **c**) The protein bands in (**a**) were quantified, and the LC3-II/GAPDH and SQSTM1/GAPDH ratios were calculated and displayed. (**d**) SJSA and MG63.2 cells were treated with CA-4 in the presence or absence of CQ, and the whole-cell lysates were subjected to immunoblotting of LC3B-II, SQSTM1 and GAPDH. (**e** and **f**) The protein bands in (**d**) were quantified, and the LC3-II/GAPDH and SQSTM1/GAPDH ratios were calculated. (**g**) SJSA and MG63.2 cells expressing GFP-LC3 were treated with control, CA-4 and CQ, and the GFP-LC3 puncta were observed under confocal microscopy. (**h**) Quantification of the number of GFP-LC3 puncta per cell in (**g**). The data were presented as mean±SD (*P⩽0.05, *n*=3)

