# Supplementary material for: NDRG1 inhibition sensitizes osteosarcoma cells to combretastatin A-4 through targeting autophagy
Source: Cell Death Dis. 2017 Sep 14;8(9):e3048–. doi: 10.1038/cddis.2017.438 (PMC5636982; doi:10.1038/cddis.2017.438)
Supplement: Supplementary Figure Legends [file cddis2017438x1.docx]

NDRG1 inhibition sensitizes osteosarcoma cells to combretastatin A-4 through targeting autophagy

**Figure Legends**

**Figure 1**. CA-4 induces autophagy in OS cells. (**A**) SJSA and MG63.2 cells were treated with various concentrations of CA-4, and the whole cell lysates were subjected to immunoblotting of LC3B-II, SQSTM1 and GAPDH. (**B**) and (**C**) The protein bands in (A) were quantified, and the LC3-II/GAPDH and SQSTM1/GAPDH ratios were calculated and displayed. (**D**) SJSA and MG63.2 cells were treated with CA-4 in the presence or absence of CQ, and the whole cell lysates were subjected to immunoblotting of LC3B-II, SQSTM1 and GAPDH. (**E**) and (**F**) The protein bands in (D) were quantified, and the LC3-II/GAPDH and SQSTM1/GAPDH ratios were calculated. (**G**) SJSA and MG63.2 cells expressing GFP-LC3 were treated with control, CA-4 and CQ, and the GFP-LC3 puncta were observed under confocal microscopy. (**H**) Quantification of the number of GFP-LC3 puncta per cell in (G). The data was presented as mean ± S.D (**P* < 0.05, n=3).

**Figure 2.** Combination treatment of CA-4 and CQ enhances anti-tumor activity in OS cells. (**A**) SJSA and MG63.2 cells treated with CA-4 alone, CQ alone, or CA-4/CQ in combination. Cell viability was determined by cell viability assay. (**B**) CA-4/CQ combination induced significantly synergistic effect in SJSA and MG63.2 cells by using CalcuSyn software. (**C**) SJSA and MG63.2 were co-treated with CA-4 and CQ, and apoptosis was indicated as sub-G1 population detected by flow cytometry. Bar graphs showed the percentages of sub-G1. (**D**) Cells were treated as in (C), and the whole cell lysates were subjected to immunoblotting of cleaved PARP, cleaved caspases, and GAPDH. (**E**) The protein bands in (D) were quantified and normalized according GAPDH. The data was presented as mean ± S.D (**P* < 0.05, n=3).

**Figure 3.** CA-4 and CQ upregulate NDRG1 expression respectively in OS cells. (**A**) SJSA and MG63.2 cells were treated with CA-4, and the whole cell lysates were subjected to immunoblotting of NDRG1 and GAPDH. **(B)** The NDRG1 double bands in (A) were quantified and normalized according GAPDH. (**C**) SJSA and MG63.2 cells were treated with CA-4, and the total mRNA was extracted. Real-time PCR was performed to determine changes in *NDRG1* mRNA. *GAPDH* was used as a loading control. (**D**) The *NDRG1* promoter-driven luciferase reporter was transfected into MG63.2 cells. The results are presented as *NDRG1* promoter activity relative to control (relative *NDRG1* promoter activity). (**E**) SJSA and MG63.2 cells were treated with CQ, and the whole cell lysates were subjected to immunoblotting of NDRG1 and GAPDH. (**F**) The NDRG1 double bands in (E) were quantified and normalized according GAPDH. (**G**) Control and CQ treated OS cells were exposed to 50 µM cycloheximide (CHX), a protein synthesis inhibitor. Cells were harvested at the indicated times (0 - 8 h) after treatment and analyzed by immunoblotting for NDRG1 and GAPDH. (**H**) NDRG1 levels in (C) were quantified and normalized to GAPDH levels, and half-life of NDRG1 was determined by regression analysis. The data was presented as mean ± S.D (**P* < 0.05, n=3).

**Figure 4**. NDRG1 knockdown inhibits autophagosome-lysosome fusion in OS cells. (**A**) MG63.2 cells were transfected with control nontarget or NDRG1 siRNA, and the whole cell lysates were subjected to immunoblotting. The lower panel shows quantitation of NDRG1 double bands. (**B**) MG63.2 cells transfected with control nontarget or NDRG1 siRNA either cultured under free-serum starved or CQ treatment, and the whole cell lysates were subjected to immunoblotting of LC3B-II and GAPDH. (**C**) The LC3-II bands in (B) were quantified and normalized according GAPDH. (**D**) and (**E**) MG63.2 cells stably expressing the mCherry-GFP-LC3 reporter were transfected with control nontarget or NDRG1 siRNA. The co-localization of mCherry and GFP puncta were examined by the confocal microscopy. (**F**) and (**G**) NDRG1 knockdown induced the accumulation of autophagic vacuoles as shown in the electron micrographs. The arrow indicates autophagic vacuoles. (**H**) Control nontarget and NDRG1 siRNA treated MG63.2 cells stably expressing GFP-LC3, and stained with antibodies against LAMP1 for confocal microscopy. (**J**) Quantification of average GFP-LC3 puncta per cell. (**K**) Quantification of GFP-LC3/LAMP1 co-localization co-efficiency. The data was presented as mean ± S.D (**P* < 0.05, n=3).

**Figure 5**. NDRG1 knockdown impairs lysosomal function in OS cells. (**A**) MG63.2 cells were stained with NDRG1 and LAMP1 antibodies in the presence or absence of CQ. The co-localization of NDRG1 and LAMP1 was examined by the confocal microscopy, scale bars: 10 µm. (**B**) Immunoblotting analysis of LAMP1 in MG63.2 cell with NDRG1 knockdown. (**C**) and (**D**) NDRG1 knockdown interferes with the acidification of lysosomes. MG63.2 cells transfected with control nontarget or NDRG1 siRNA were exposed to LysoTracker for confocal microscopy. (**E**) Lysosomal pH values were measured using a quantitative ratiometric LysoSensor Yellow/Blue DND-160.  Scale bars: 500 nm. (**F**) Enzymatic activity of CTSB was measured in MG63.2 cells transfected with control nontarget or NDRG1 siRNA using fluorogenic kits. (**G**) MG63.2 cells transfected with control or NDRG1 siRNA were treated with 50 ng/ml EGF, and EGFR, p-AKT and p-ERK were determined at each point by immunoblotting. (**H**) The EGFR bands in (G) were quantified and normalized according GAPDH. The data was presented as mean ± S.D (**P* < 0.05, n=3).

**Figure 6**. NDRG1 knockdown sensitizes CA-4 and CQ combination treatment-induced apoptosis in OS cells. (**A**) Cells transfected with control nontarget or NDRG1 siRNA treated with CA-4 alone, CQ alone, or CA-4/CQ in combination. Cell viability was determined by cell viability assay. (**B**) Apoptosis was indicated as sub-G1 population detected by flow cytometry. (**C**) Cells were harvested, and then the whole cell lysates were subjected to immunoblotting of cleaved PARP, cleaved caspase 3, and GAPDH. (**D**) The cleaved PARP and cleaved caspase 3 bands in (C) were quantified and normalized according GAPDH. The data was presented as mean ± S.D (**P* < 0.05, n=3).

**Figure 7.** Model of the role of NDRG1 in the CA-4-induced apoptosis and autophagy. Down-regulation of NDRG1 expression causes the defect of lysosomal function, subsequently resulting in decreasing the fusion between autophagosomes and lysosomes. Moreover, NDRG1 inhibition increases apoptosis in response to combination treatment with CA-4 and CQ.

**Supplymentary Figure Legends**

**Figure S1.** (**A**) CCHO, 143B and K7 cells were treated with various concentrations of CA-4. The whole cell lysates were subjected to immunoblotting of LC3B-II and GAPDH.

**Figure S2. (A)** and **(B)** SJSA and MG63.2 cells treated with CA-4, or CQ in combination. Cell viability was determined by cell viability assay. **(C)** CCHO, 143B, K7 and Dunn cells treated with CA-4 alone, CQ alone, or CA-4/CQ in combination. Cell viability was determined by cell viability assay. The data was presented as mean ± S.D (**P* < 0.05, ***P* < 0.01, n=3).

**Figure S3.** **(A)** SJSA and MG63.2 cells were treated with CQ, and the total mRNA was extracted. Real-time PCR was performed to determine changes in *NDRG1* mRNA. (**P* < 0.05, n=3).

**Figure S4.** (**A**) SJSA cells stably expression GFP-LC3 was transfected with control nontarget or NDRG1 siRNA either cultured under normal growth conditions or free-serum starved for 12 h. The GFP-LC3 puncta were observed under confocal microscopy.

**Figure S5.** (**A)** MG63.2 cells transfected with control nontarget or NDRG1 siRNA were exposed to 100 nM LysoTracker for flow cytometry. (**B**) MG63.2 cells transfected with control nontarget or NDRG1 siRNA were exposed to 100 nM LysoTracker for confocal microscopy. Scale bars: 500 nm.
