# Supplementary figures and images for: NDRG1 inhibition sensitizes osteosarcoma cells to combretastatin A-4 through targeting autophagy
Source: Cell Death Dis. 2017 Sep 14;8(9):e3048–. doi: 10.1038/cddis.2017.438 (PMC5636982; doi:10.1038/cddis.2017.438)

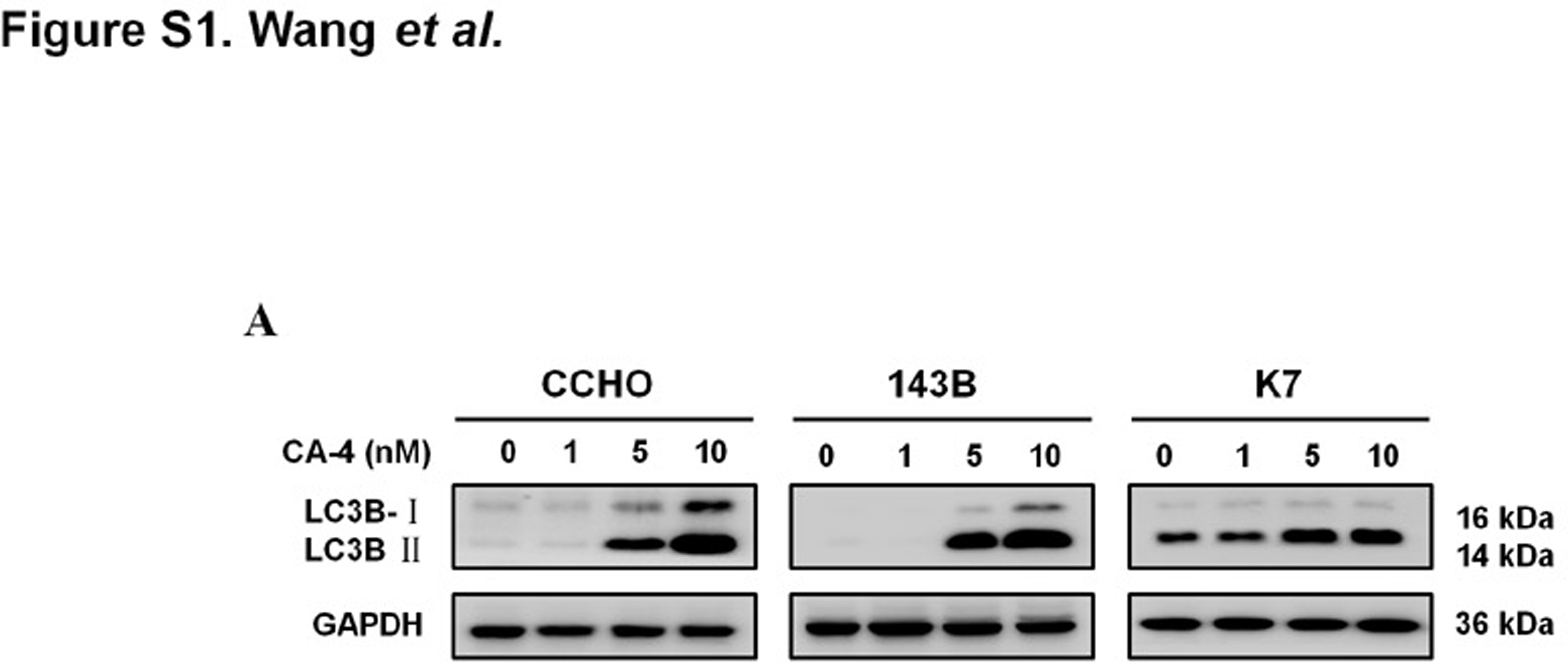

Supplement: Supplementary Figure 1 [file cddis2017438x2.tif]

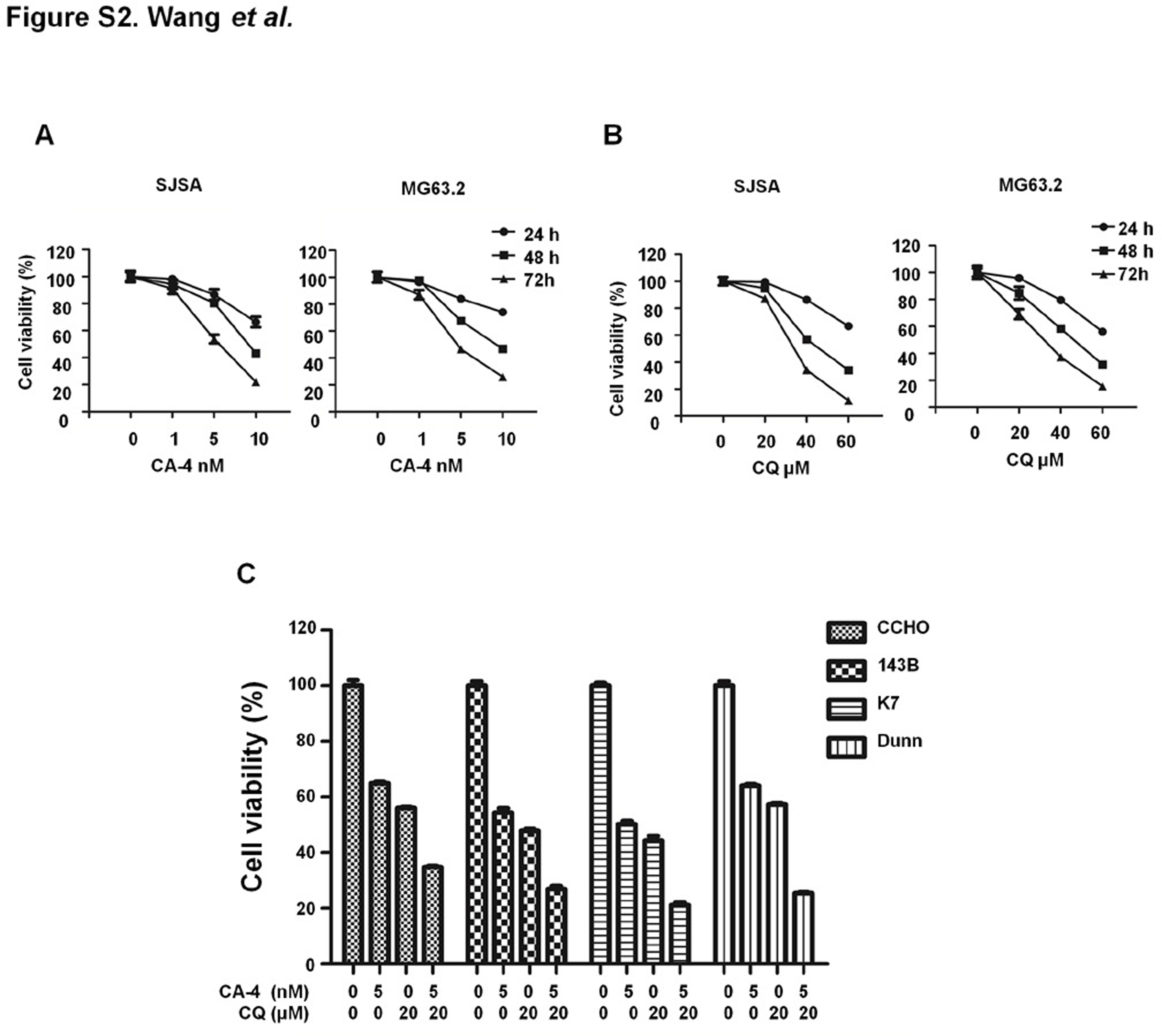

Supplement: Supplementary Figure 2 [file cddis2017438x3.tif]

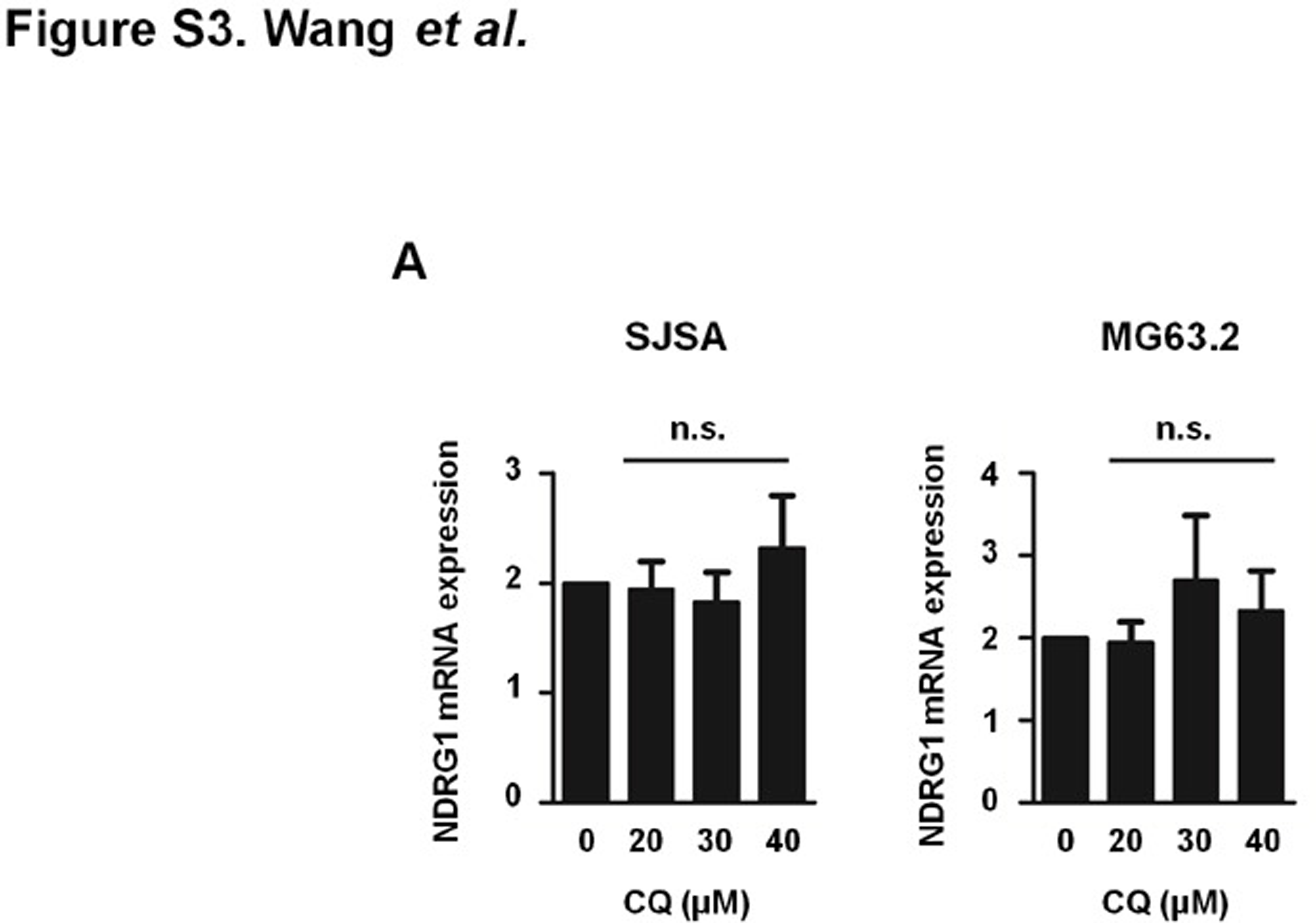

Supplement: Supplementary Figure 3 [file cddis2017438x4.tif]

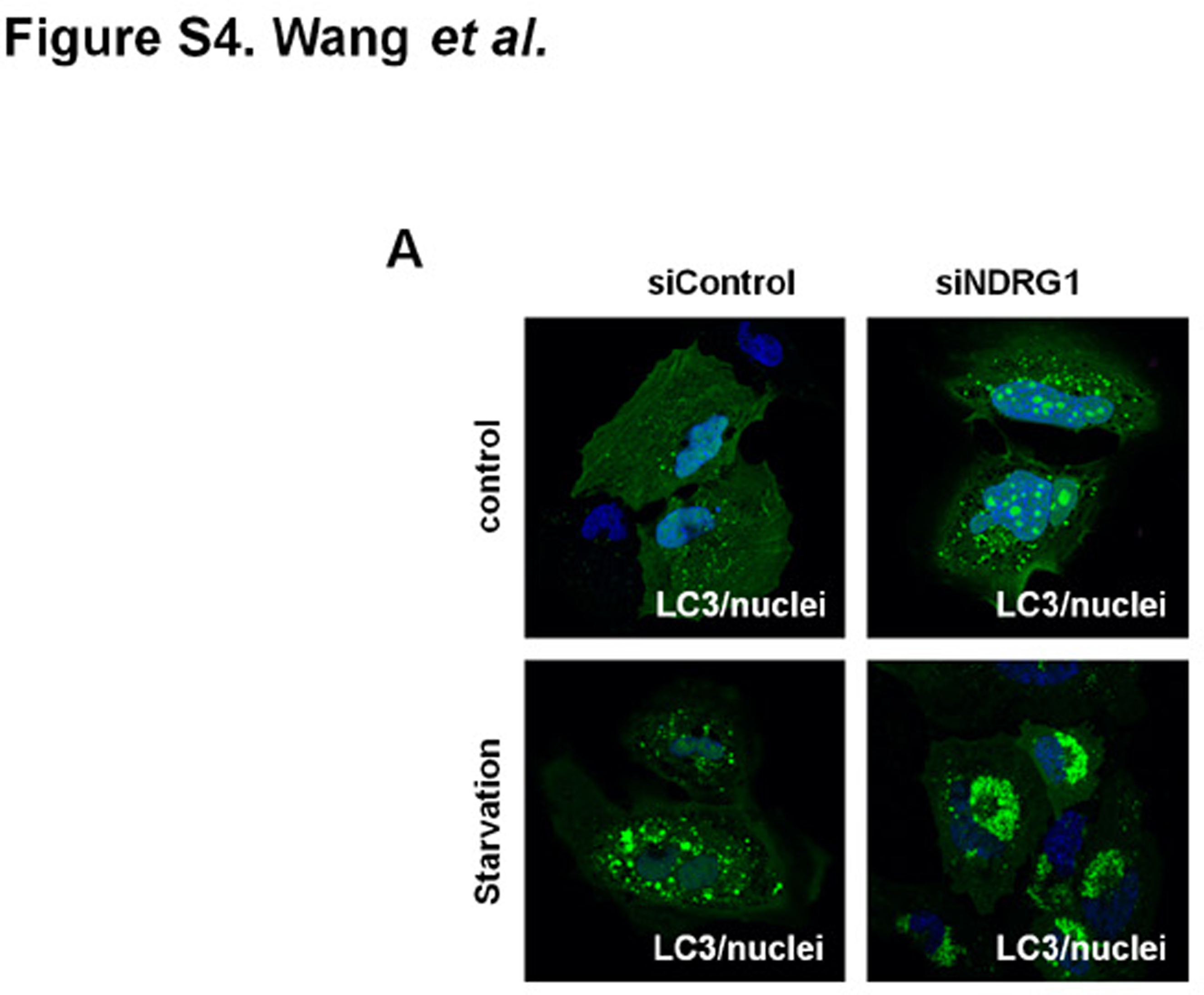

Supplement: Supplementary Figure 4 [file cddis2017438x5.tif]

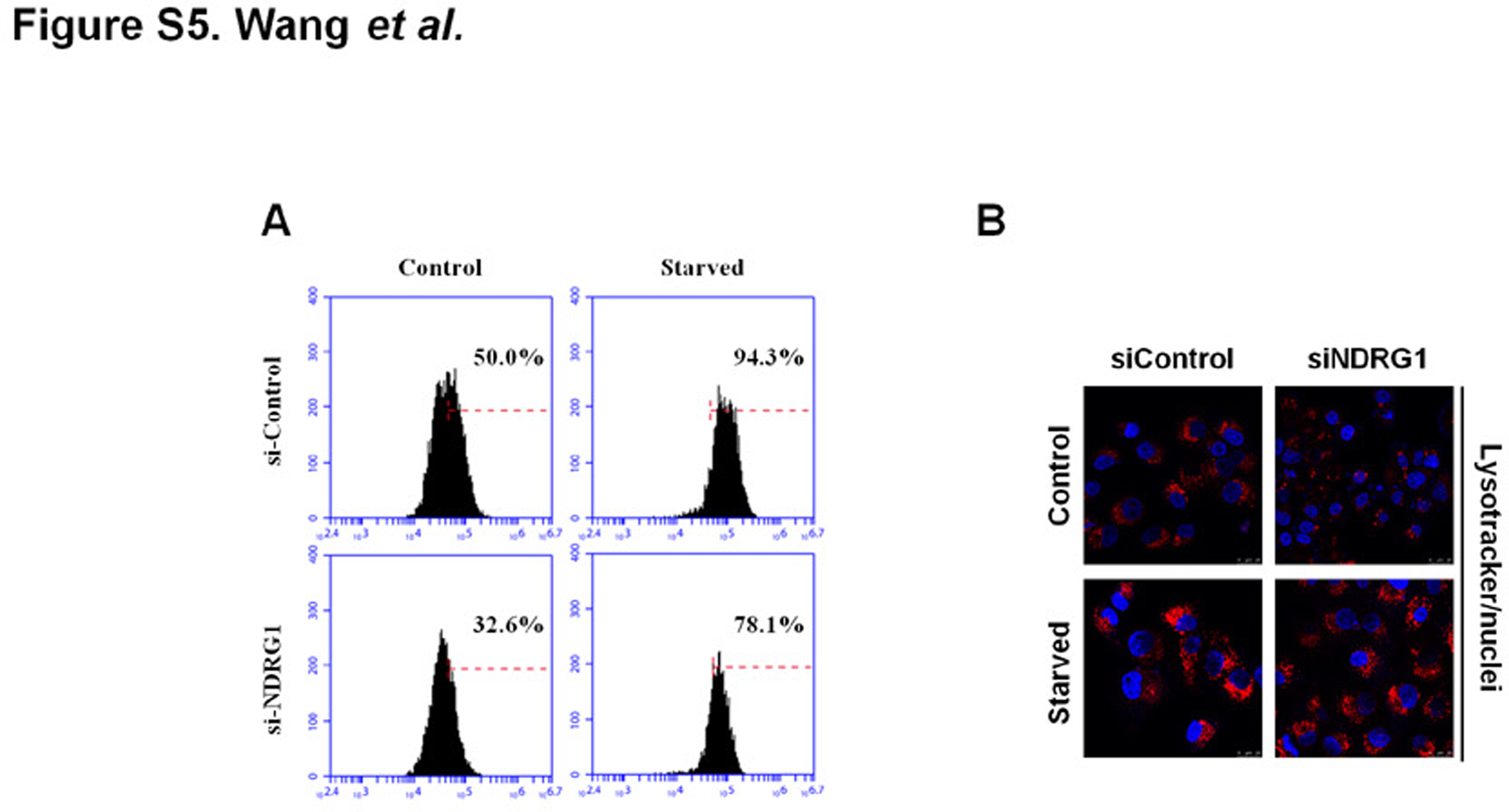

Supplement: Supplementary Figure 5 [file cddis2017438x6.tif]
